# Fatal Crossroads: D-penicillamine and Disseminated Intravascular Coagulation in Wilson’s Disease

**DOI:** 10.7759/cureus.80726

**Published:** 2025-03-17

**Authors:** Razan Mando, Hamza Haj Mohamad, Mariam Abdelkader, Amna Alketbi, Mohammed Alzebari, Dhuha Ali Eledresi, Ubaid Hashmi, Mahasin Shaheen

**Affiliations:** 1 School of Medicine, Al Qassimi Hospital, Sharjah, ARE; 2 Medicine, University of Sharjah, Sharjah, ARE; 3 School of Medicine, Burjeel Medical City, Abu Dhabi, ARE; 4 Emergency Medicine, Mohammed Bin Rashid University, Sharjah, ARE; 5 Medicine, Al Qassimi Hospital, Sharjah, ARE; 6 Internal Medicine, Al Qassimi Hospital, Sharjah, ARE

**Keywords:** dic, disseminated intravascular coagulation, d-penicillamine, liver failure, wilson’s disease

## Abstract

A 13-year-old male with Wilson’s disease presented with progressive edema, jaundice, and neuropsychiatric symptoms. His evaluation revealed advanced hepatic dysfunction (Child-Pugh Class C), confirmed by biochemical, imaging, and histopathological findings. Despite initial stabilization, he was readmitted with acute gastrointestinal bleeding, hypotension, and laboratory evidence of disseminated intravascular coagulation (DIC), which rapidly progressed despite aggressive management. This case underscores the potential risk of DIC with D-penicillamine in Wilson’s disease and highlights the need for vigilance, early recognition, and consideration of alternative treatments in susceptible patients.

## Introduction

Disseminated intravascular coagulation (DIC) is a systemic disorder characterized by uncontrolled activation of coagulation and fibrinolysis, leading to widespread microvascular thrombi, consumption of clotting factors, and a paradoxical risk of both thrombosis and hemorrhage. It is commonly associated with malignancy, sepsis, and trauma but can also result from liver disease, pancreatitis, transfusion reactions, and surgery [1]. 

Wilson’s disease is an autosomal recessive disorder caused by *ATP7B* gene mutations, leading to copper metabolism dysfunction. D-penicillamine is an effective treatment for Wilson’s disease; however, it has been associated with hematologic complications, including thrombocytopenia, hemolytic anemia, and thrombotic thrombocytopenic purpura [2]. Here, we present a case of disseminated intravascular coagulation as a complication of D-penicillamine use in Wilson’s disease.

## Case presentation

A 13-year-old male presented to the hospital with a four-day history of progressive edema involving both lower limbs, hands, and face. His past medical history was notable for recurrent admissions due to hepatosplenomegaly, ascites, and severe anemia necessitating multiple blood transfusions. His family reported recent changes in behavior, including increased aggression and episodes of disorientation. They also observed worsening jaundice, particularly a yellow discoloration of his sclera. He denied any fever, vomiting, nausea, abdominal pain, or changes in bowel habits.

On admission, his vital signs were within normal limits: temperature of 36.7°C, heart rate of 82 beats per minute, respiratory rate of 18 breaths per minute, blood pressure of 120/70 mmHg, and oxygen saturation of 99% in room air. Physical examination revealed a conscious but agitated and aggressive patient. His abdomen was soft, lax, and non-tender, with significant hepatosplenomegaly. Notable edema was observed in the bilateral upper and lower extremities, as well as in the face. Laboratory findings on admission are shown in Table 1.

**Table 1:** Key laboratory findings for a 13-year-old male diagnosed with Wilson’s disease. Table including relevant markers for hepatic function, copper metabolism, hematologic status, and associated abnormalities. These values contribute to the assessment of the patient's Child-Pugh score and overall liver function.

Test name	Value	Normal range	Significance
Total bilirubin	118.5 µmol/L	3.0-17.0 µmol/L	Elevated; indicates severe hepatic dysfunction, contributes to Child-Pugh score.
Albumin	21 g/L	38.0-56.0 g/L	Low; reflects impaired liver synthetic function, contributes to Child-Pugh score.
Prothrombin time (PT)	29.80 seconds	12.5-15.2 seconds	Prolonged PT/INR indicates coagulation impairment, included in Child-Pugh score.
Internalized normalized ratio (INR)	2.72	0.80-1.29	High; further indication of hepatic dysfunction.
Aspartate transferase (AST)	104 IU/L	15-37 IU/L	Elevated; indicates hepatocellular damage.
Alkaline phosphatase	1643.6 IU/L	116-468 IU/L	Significantly elevated; may suggest biliary involvement.
Serum copper	299.7 µg/L	57-129 µg/dL	Elevated; supports the diagnosis of Wilson’s disease.
Ceruloplasmin	0.04 g/L	0.15-0.30 g/L	Low; a key marker for Wilson’s disease.
Copper/creatinine ratio	230.9 µg/g	Up to 13.1 µg/g	Elevated; indicative of excess urinary copper excretion.
Hemoglobin	10.8 g/dL	13.0-17.0 g/dL	Low; possible effect of splenomegaly and portal hypertension.
Platelet count	65,000/µL	150,000-450,000/µL	Low; likely related to hypersplenism.
Ammonia	101 µmol/L	11-32 µmol/L	Elevated; correlates with neurological symptoms and hepatic encephalopathy.
C-reactive protein (CRP)	9.6 mg/L	0-3 mg/L	Mildly elevated; may indicate inflammatory response.
Total creatine kinase (CK)	>1000 U/L	Up to 250 U/L	High; suggests muscle damage, though not directly related to Wilson’s disease.

**Figure 1:**
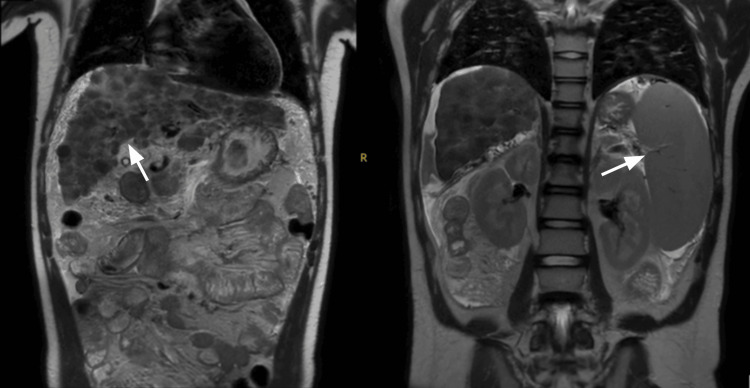
MRI scan of the abdomen showing irregular nodular surface of the liver, with hepatomegaly (left) and splenomegaly (right)

His liver function assessment indicated a Child-Pugh score of 12, classifying him as Child-Pugh Class C, suggestive of decompensated liver disease. Abdominal ultrasound findings showed a cirrhotic liver with thickening of the gallbladder wall without cholelithiasis, marked splenomegaly, and a small amount of free fluid in the pelvis. An esophagogastroduodenoscopy showed portal hypertensive gastropathy in the fundus and body with one small varix. Campylobacter-like organism (CLO) test, a rapid urease test, was negative. A liver biopsy revealed hepatic fibrosis with nodular regeneration. Notably, a slit-lamp examination identified Kayser-Fleischer rings, a hallmark of Wilson’s disease. This constellation of clinical findings, imaging, and slit-lamp findings pointed to a diagnosis of Wilson’s disease with advanced hepatic involvement. A magnetic resonance imaging scan shows an irregular nodular surface of the liver, with associated splenomegaly and ascites (Figure 1).

After discussion with a gastroenterologist, the patient’s management plan included lactulose syrup 30 mL thrice daily for hepatic encephalopathy; carvedilol 3.125 mg twice daily, Rifaximin 550 mg thrice daily, and Ursodiol 250 mg thrice daily for liver cirrhosis; Penicillamine 250 mg every 6 hours and Zinc acetate 50 mg twice daily for management of Wilson’s Disease. He also received continuous intravenous fluids (0.9% sodium chloride). His at-home medications, diuretics (furosemide 20 mg daily, spironolactone 12.5 mg daily), and a proton pump inhibitor (pantoprazole 40 mg daily) were also continued. The patient was discharged on these medications and scheduled for a follow-up with the gastroenterologist to prepare for a liver transplant. 

The patient was readmitted two weeks after discharge to the emergency department (ED) with acute gastrointestinal symptoms, including multiple episodes of diarrhea and vomiting, initially containing food, then progressing to threads of blood. His mother noted a rapid onset of fatigue, back pain, and fever, all of which intensified over two hours. On arrival, he exhibited physiological instability, with a Glasgow Coma Scale (GCS) score of 10, tachycardia at 126 beats per minute, tachypnea at 24 breaths per minute, and hypotension (85/40 mmHg). Lab results revealed significant hepatic dysfunction, including elevated internalized normalized ratio (INR), prolonged bleeding and clotting times, and abnormal liver function tests, indicative of acute hepatic decompensation.

**Figure 2:**
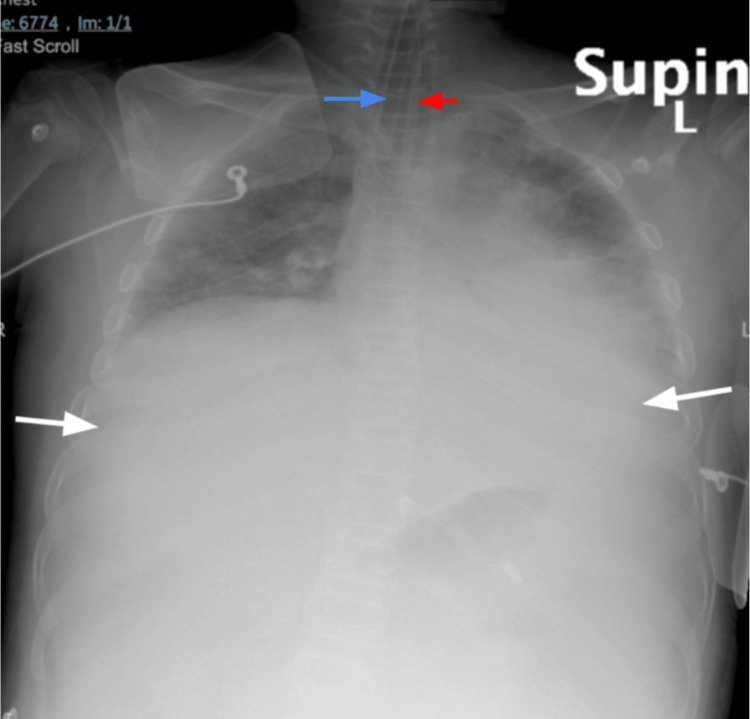
Supine chest X-ray showing pulmonary edema (white arrows), endotracheal tube (blue arrow), nasogastric tube (red)

An initial nasogastric tube (NGT) placement produced coffee ground material, indicating an upper gastrointestinal bleed, though the focus remained on stabilizing his critical hypotension and addressing abnormal lab findings. Suspected acute fulminant liver failure necessitated urgent evaluation for a liver transplant, and a full septic workup was initiated. A supine X-ray confirmed the presence of the newly inserted endotracheal tube (blue arrow) and nasogastric tube (red arrow) in place. It also shows pulmonary edema bilaterally (white arrows) (Figure 2). 

To manage his hemodynamic instability, the patient received 30 micrograms of noradrenaline, along with antibiotics and symptomatic treatment, including lactulose and a continuous pantoprazole infusion. Despite these measures, he deteriorated rapidly; his Glasgow Coma Score dropped to 7, prompting intubation. His blood glucose was critically low at 1.8 mmol/L, and his blood pressure remained unstable at 90/70 mmHg despite increased inotropic support.

Lab results soon indicated disseminated intravascular coagulation (DIC) and acute renal failure, with internalized normalized ratio (INR)>6, platelets at 22, aspartate transferase (AST) 236 IU/L, alanine transaminase (ALT) 74 IU/L, severe hypofibrinogenemia at 30mg/dL (normal 100 mg/dL) and serum creatinine 216 µmol/L, alongside anuria. In response, he received six units of fresh frozen plasma (FFP), albumin, and sodium chloride boluses, with two units of packed red blood cells (PRBCs) on standby. Intravenous vitamin K was initiated to aid coagulation. D-penicillamine 1 gram/day divided to four times daily was initiated.

The next day, he developed extensive spontaneous bleeding from multiple sites, confirming disseminated intravascular coagulation, with hemoglobin dropping from 11 to 7 g/dL and worsening coagulation values. Despite aggressive transfusion support including packed red blood cells, fresh frozen plasma, cryoprecipitate, tranexamic acid, vasopressin, and sodium bicarbonate, his blood pressure fell to a critical 60/30 mmHg. Given his condition and lack of transplant access, the team informed his family of the grave prognosis. Sadly, despite 30 minutes of cardiopulmonary resuscitation, the patient could not be resuscitated and was pronounced deceased.

## Discussion

Disseminated intravascular coagulation (DIC) is a consumptive coagulopathy characterized by a homeostatic imbalance between coagulation and fibrinolysis. Triggered by factors such as tissue damage or systemic inflammation, disseminated intravascular coagulation begins with the activation of tissue factor and coagulation factor VIIa, leading to thrombin and fibrin formation. This process generates widespread microvascular clots, consuming platelets and coagulation factors, while dysregulation of inhibitory pathways like the protein C system and antithrombin III exacerbates bleeding and clotting tendencies [1].

Wilson’s disease, on the other hand, arises from mutations in the *ATP7B* gene, impairing copper metabolism and leading to copper accumulation in the liver and subsequent release into the bloodstream. Excess copper triggers oxidative stress and cellular damage, manifesting as liver dysfunction, neuropsychiatric symptoms, and systemic complications [2]. In advanced stages, the liver’s impaired synthetic function and heightened inflammation can create a procoagulant environment, linking Wilson’s disease to potential complications like disseminated intravascular coagulation.

In our patient, the convergence of hepatic dysfunction, systemic inflammation, and oxidative stress in Wilson’s disease could have contributed to disseminated intravascular coagulation development. The International Society on Thrombosis and Hemostasis (ISTH) score of 5 (overt disseminated intravascular coagulation) is corroborated by prolonged prothrombin time, severe hypofibrinogenemia, and thrombocytopenia, providing a clear diagnostic framework for this consumptive coagulopathy.

While Wilson’s disease can predispose patients to disseminated intravascular coagulation, the timing of this complication following the initiation of D-penicillamine therapy suggests a potential causal role for the drug. D-penicillamine, a cornerstone treatment for Wilson’s disease, has been linked to rare cases of disseminated intravascular coagulation through mechanisms such as immune dysregulation, hypersensitivity reactions, or exacerbation of hepatic dysfunction. In our patient, the onset of disseminated intravascular coagulation just two weeks after starting D-penicillamine supports this hypothesis.

In 2022, a case study was conducted on a 24-year-old female with Wilson’s disease who developed disseminated intravascular coagulation during D-penicillamine treatment, presenting with mucosal bleeding and coagulopathy resistant to plasmapheresis and factor replacement. Despite managing hepatic encephalopathy and bilirubin elevation, her coagulopathy and bleeding persisted until D-penicillamine was replaced with trientine. Following this change, her mucosal bleeding ceased, disseminated intravascular coagulation parameters improved, and there was no further need for factor replacement over the next four months [3].

Apart from an animal study, no research has explored the potential for D-penicillamine to induce disseminated intravascular coagulation during long-term use. In that study, rats treated with D-penicillamine developed disseminated intravascular coagulation and subsequently died [4]. Proposed mechanisms for disseminated intravascular coagulation development include endothelial damage caused by D-penicillamine, leading to the exposure of subendothelial collagen and triggering intravascular coagulation via collagen-reactive platelets [5]. Additionally, endothelial injury may stimulate the release of platelet-activating factors, thromboxane A2, or other prostaglandin-like substances, contributing to the onset of disseminated intravascular coagulation [5].

If D-penicillamine is indeed implicated in the development of disseminated intravascular coagulation in Wilson’s disease, this raises critical questions about its safety in patients with pre-existing hepatic or coagulation abnormalities. Early recognition of disseminated intravascular coagulation symptoms and immediate discontinuation of the drug are essential. Developing standardized criteria to identify high-risk patients and determine when to withhold or discontinue D-penicillamine in the context of disseminated intravascular coagulation is imperative. Alternative treatments, such as trientine or zinc acetate, may offer safer options in such scenarios.

## Conclusions

In conclusion, this case highlights the potential for D-penicillamine to exacerbate coagulopathy in patients with Wilson's disease and advanced hepatic dysfunction. Clinicians should carefully assess the risk-benefit balance before initiating therapy, particularly in patients with pre-existing coagulopathy. Alternative chelators such as trientine may warrant consideration in high-risk individuals. Further research into the mechanisms linking D-penicillamine to coagulopathy could provide valuable insights for refining treatment protocols. Ultimately, early recognition and prompt intervention are crucial in mitigating life-threatening events in patients with Wilson disease receiving chelation therapy.
